# Clearance of dying cells accelerates malignancy

**DOI:** 10.18632/oncotarget.5670

**Published:** 2015-09-15

**Authors:** David B. Vaught, Rebecca S. Cook

**Affiliations:** Department of Cancer Biology, Vanderbilt University and the Vanderbilt Ingram Cancer Center, Nashville, TN, USA

**Keywords:** efferocytosis, MerTK, macrophages, post-partum breast cancer, tumor microenvironment

The breast endures vast changes during reproductive phases of a woman's life (puberty, pregnancy, lactation, post-partum involution, post-menopausal involution). Each phase uniquely shapes cancer susceptibility, formation, and progression. Although pregnancy at a young age decreases lifetime breast cancer risk, the first five years following pregnancy at any age are associated with increased breast cancer risk regardless of the woman's age, and with even greater risk with increasing age at the woman's first pregnancy [1]. Increasingly, women are postponing child-birth, which may increase the incidence of post-partum breast cancer (ppBC), defined as those breast cancers diagnosed 2–5 years after pregnancy. These ppBCs are distinguishable from those breast cancers that are diagnosed and treated during pregnancy, and which never are exposed to post-partum/post-lactational involution, and which correlate with a favorable prognosis. Currently, ppBC accounts for nearly 25% of all breast cancers in young (pre-menopausal) women. In contrast, ppBCs are highly aggressive, metastatic, and life-threatening, even when corrected for molecular breast cancer subtype and for the age of the woman at diagnosis [1].

Mouse models of ppBC that specifically compare mammary tumors from nulliparous (virgin) mice to those from age-matched parous (single pregnancy) mice confirm that post-partum involution increases metastasis by up to 10-fold [2, 3]. The molecular mechanisms underlying the exaggerated lethality of post-partum breast cancers are related to an exaggerated abundance of M2-like tumor associated macrophages, which produce immune suppressive and wound healing cytokines and proteases that modify the post-partum mammary (and tumor) microenvironment [4], although the mechanisms that trigger this shift in macrophage behavior in the post-partum mammary gland remained obscure. It was recently demonstrated that widespread cell death, a hallmark of the mammary gland during post-partum involution when milk production ceases, triggers macrophage-mediated efferocytosis, M2 macrophage polarization and Th2 cytokine production in normal mammary glands during post-partum involution [5]. Remarkably, widespread cell death efferocytosis, macrophage M2 polarization, and Th2 cytokine-mediated wound healing in malignant post-partum breast cancers was similarly observed [3].

Under physiological conditions, dying cells are rapidly removed from the breast to prevent secondary necrosis of the dying cell, wherein intracellular antigens released from the necrotic cell might trigger inflammation, tissue damage, or autoimmunity [6]. To ensure suppression of inflammation or autoimmunity, efferocytosis is coupled with production of cytokines that dampen tissue-damaging immune responses, such as interleukin (IL)-10, IL-4, and Transforming Growth Factor (TGF)-β [7]. Macrophages use multiple cell surface protein to recognize and engulf dying cells. Among these, the receptor tyrosine kinase (RTK) MerTK is essential for post-partum efferocytosis and for subsequent induction of immunosuppressive and wound healing cytokines [6]. Genetically engineered mouse models lacking MerTK activity display impaired efferocytosis and limited expression of wound healing cytokines during post-partum involution, resulting in severe immune-mediated damage and scarring to the post-partum mammary gland that interferes with the success of lactogenesis upon future pregnancies [5].

We recently found that efferocytosis was a key driver of malignant progression in ppBCs, responsible for exaggerated M2-like polarization of tumor-infiltrating macrophages and production of IL-4, TGFβ, and IL-10 [3]. Genetic MerTK ablation inhibited efferocytosis in ppBCs, blocked macrophage M2-like polarization, impaired expression of efferocytosis-induced cytokines, and repressed formation of lung metastases. Pharmacologic inhibition of MerTK for the first 7 days of post-partum involution similarly blocked efferocytosis, and significantly decreased metastatic burden. Thus, a causal relationship exists between the tissue remodeling during physiological postpartum involution and the increased metastasis of postpartum mammary tumors. Both scenarios are characterized by transient and widespread programmed cell death, efferocytosis, and the abundant M2-like macrophages and wound-healing cytokines that associate with reduced breast cancer survival.

These observations highlight tumor cell death as a double-edged sword in the tumor microenvironment: although the chemotherapies, targeted therapies and radiation provide the benefit of widespread tumor cell death and tumor shrinkage, widespread efferocytosis in response to tumor cell death may enhance tumor wound healing, thus limiting the effectiveness of the targeted agent. In some cases, efferocytosis may even promote tumor metastasis. These issues require careful consideration and experimental testing, as the role of efferocytosis in modulating the stromal response to therapeutically-induced tumor cell death is not fully understood. These recent findings support future endeavors to examine efferocytosis/MerTK targeting in combination with current treatment strategies to block unhealthy ‘tumor healing’ and improve tumor response to treatment.
